# Variations of Median Nerve Formation in North Indian Population

**DOI:** 10.7759/cureus.20890

**Published:** 2022-01-03

**Authors:** Jigyasa Passey, Pareesa Rabbani, Shayama K Razdan, Shalini Kumar, Arvind Kumar

**Affiliations:** 1 Department of Anatomy, Hamdard Institute of Medical Sciences and Research, New Delhi, IND; 2 Department of Orthopaedics, Hamdard Institute of Medical Sciences and Research, New Delhi, IND

**Keywords:** upper limb, triple roots, median nerve, dissection, brachial plexus, anatomy

## Abstract

Introduction: The median nerve is usually formed by two roots contributed from the medial and lateral cords of the brachial plexus. Morphological variations of the median nerve can have clinical implications from the anesthetic and surgical points of view. In this cadaveric observation study, we report the variations of median nerve formation in the North Indian population.

Methods: We observed the formation of the median nerve in 40 human cadaveric upper limb specimens. The specimens belonged to 20 right and 20 left upper limbs. Variations in the formation of the median nerve were noted.

Results: Of the 40 dissected specimens, six (15%) had triple roots including a supernumerary root contributing to the medial nerve formation. The supernumerary root was a branch of the lateral cord in five cases, and it had an additional contribution from the medial cord in one case. The median nerve formation and continuation were located anterior or laterally in 39 specimens (97.5%) and medial in one (2.5%) in relation to the axillary artery.

Conclusion: We observed supernumerary roots of varying morphology contributing to the median nerve formation. These variations should be considered during the administration of regional anesthesia and during the management of brachial plexus injuries. Further large multi-region studies will help in a better understanding of these variations.

## Introduction

The muscles, joints, and skin of the upper limb are innervated by the brachial plexus formed by the contributions from the ventral primary rami of C5, C6, C7, C8, and T1 nerve roots. A complex exchange of interconnections produces the brachial plexus's trunks, divisions, cords, and branches. As per Gray's Anatomy [1], the formation and relations of the three cords of the brachial plexus are variable. They assume their befitting relations around the axillary artery deep to pectoralis minor. Two roots usually form the median nerve contributed by the medial and lateral cords of the brachial plexus. The greatest variation related to median nerve formation lies in the lateral cord, where the lateral root of the median nerve may arise as two or three heads [2,3]. Morphological variations of the median nerve can have clinical implications from the anesthetic and surgical points of view. Awareness of such variations can be useful for preventing iatrogenic injuries during surgical and anesthetic procedures around the axilla. In this cadaveric observational study, we report the variations of median nerve formation in the North Indian population.

## Materials and methods

The study was conducted in a tertiary care teaching institute in North India. Forty formalin-fixed human upper limb cadaveric specimens (20 left, 20 right) were dissected according to Cunningham’s Manual of Practical Anatomy. The brachial plexus in each specimen was exposed and studied meticulously to determine the anatomical variations of the formation of the median nerve. We made observations regarding the median nerve’s number of roots, their gross morphology, and the median nerve formation in relation to the axillary artery. The thickness of any additional root was measured using a digital vernier caliper. Photographs were taken and labeled. The collected data were charted and compared with the available evidence in the literature.

## Results

**Figure 1:**
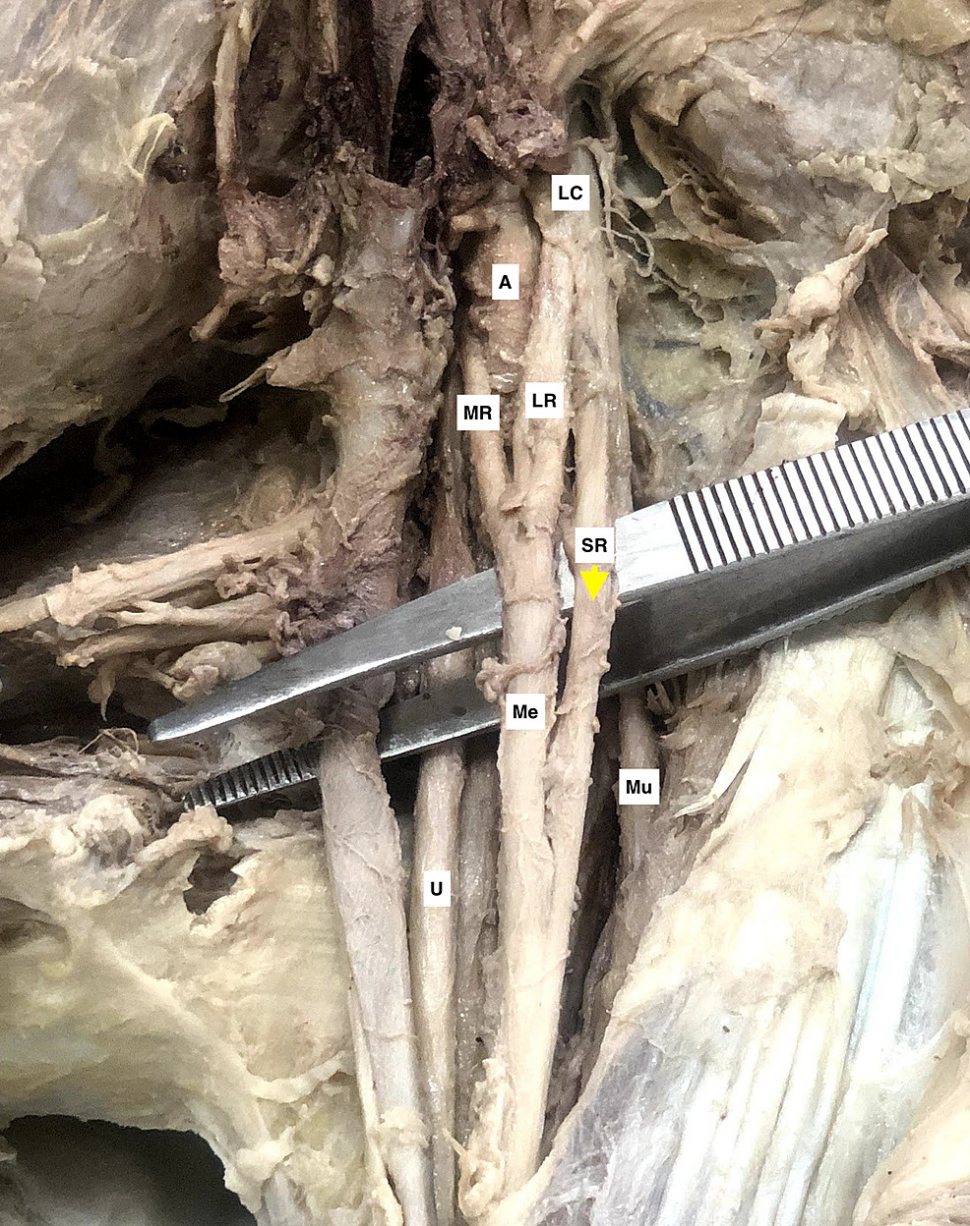
A cadaveric specimen of left axilla with brachial plexus showing a thick tubular supernumerary root (SR, yellow arrowhead) originating from the lateral aspect of the lateral cord (LC) and blending distally with the median nerve (M) after its formation. The axillary artery (A), lateral root (LR), and medial root (MR) of the median nerve (M), musculocutaneous nerve (Mu), and ulnar nerve (U) can be seen in the adjacent region.

**Figure 2:**
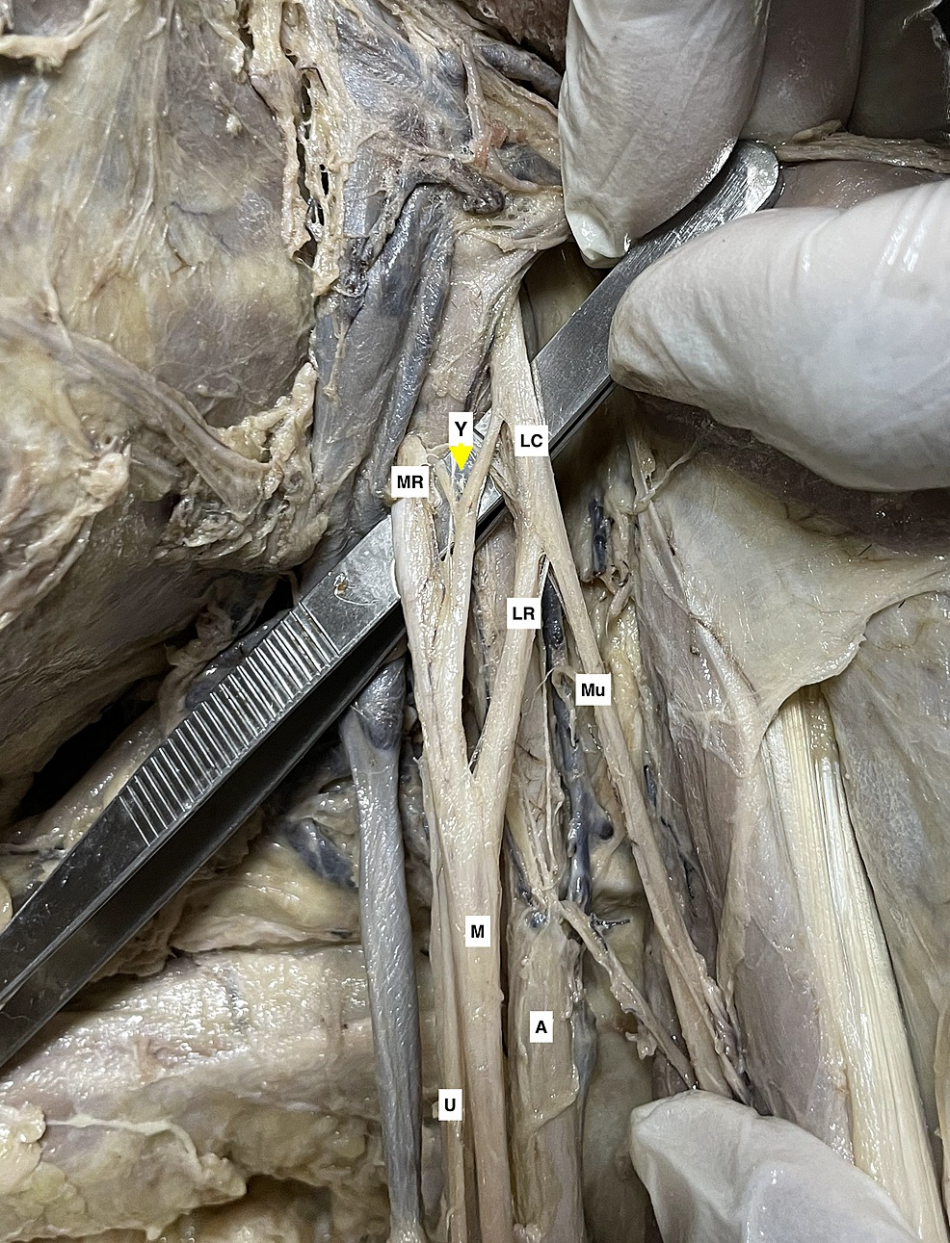
A cadaveric specimen of left axilla with brachial plexus showing a "Y" shaped supernumerary root (yellow arrowhead), arising from the lateral cord (LC) and the medial root (MR) of the median nerve (M). The axillary artery (A), lateral root (LR) of the median nerve, musculocutaneous nerve (Mu), and ulnar nerve (U) can be seen in the adjacent region.

Besides medial and lateral roots, an additional contributing root to the median nerve was observed in six cases. The supernumerary root was arising from the lateral cord alone in five cases. In one of these five cases, the supernumerary root originated from the lateral aspect of the lateral cord. It merged distally with the median nerve that was already formed (Figure 1). In the others, the supernumerary root arose from the medial aspect of the lateral cord and then contributed to the median nerve formation. We encountered another rare entity where the extra root was Y shaped with contribution from both medial and lateral cords (Figure 2). The medial and lateral contributary bands of the supernumerary root were of 1 mm thickness each. The thickness of the supernumerary roots varied from 1.2 mm to 5 mm. The length of the supernumerary roots varied from 2 cm to 4.3 cm. We encountered only one case of median nerve formation lying medial to the axillary, and it was located anterior or lateral in the remaining cases. The detailed findings are presented in Table 1.

**Table 1:** Morphometric characteristics of variations of the median nerve formation.

Specimen	Side	Number of contributing roots	The thickness of supernumerary root (mm)	Length of supernumerary root (cm)	Relation to the axillary artery (anterolateral/ medial/posterior)
Specimen 1	Left	3	1.2	2	Medial
Specimen 2	Right	3	4.2	3.2	Anterolateral
Specimen 3	Left	3	3	2.8	Anterolateral
Specimen 4	Right	3	3.1	3.4	Anterolateral
Specimen 5	Left	3	5	4.3	Anterolateral
Specimen 6	Left	3	2.4	2	Anterolateral

## Discussion

The current study highlights the variations in the formation of the median nerve in cadaveric specimens based on the North Indian population. While most specimens had the medial and lateral roots from the medial and lateral cords, we noticed additional supernumerary roots in 15% of specimens. The variations can affect invasive and noninvasive procedures around the brachial plexus. Axillary block has been commonly used for anesthesia in upper limb surgeries. The axillary approach for the brachial plexus block is the safest due to the reduced risk of injury to surrounding structures such as the phrenic nerve blockade and pneumothorax. However, the general risks of unplanned intravascular and intraneural injection persist [4]. Therefore, the brachial plexus and nerve branching variations in this area are of clinical importance. In addition, surgeons should carefully handle brachial plexus injuries presenting with injury to the median nerve and roots involved in its formation to avoid any injury to any supernumerary root. Unrepaired status of the supernumerary roots can result in a persistent unpredictable neurological deficit. An awareness of such variations would help avoid intraoperative confusion in complex injury patterns.

The etiology of median nerve formation variation can be traced to its embryonic development. The somites forming the limbs migrate and bring their respective nerve supply such that each myotome retains its basic segmental innervation [5]. The seventh cervical segmental artery forms the axillary artery and is closely related to the cords of the brachial plexus. However, at times the subclavian-axillary stem is derived from the sixth or eighth segmental arteries establishing an abnormal relation about the cords of the brachial plexus [5]. As presented in Table 2, our findings were similar to other North Indian studies [2,6]. However, a strikingly higher incidence was observed in Central Indian, Malaysia, Nepal populations, and lower incidence in Iranian and Sri Lankan populations [7-11]. The similarity with other North Indian studies may indicate ethnic predisposition of the variations. However, dissimilar results in Central Indian and other Asian descents prove a highly nonuniform incidence pattern. Few studies also report four roots of the formation of the median nerve [7], but we did not encounter any such case.

**Table 2:** Incidence of anatomical variation in the number of roots in the formation of the median nerve.

Study	Population	Year	Supernumerary root incidence
Jyoti et al. [2]	North Indian	2020	16.66%
Priya et al. [6]	North Indian	2019	13.3%
Budhiraja et al. [7]	Central Indian	2011	26%
Mat Taib et al. [8]	Malaysia	2016	29.5%
Hada et al. [9]	Nepal	2020	20%
Emamhadi et al. [10]	Iran	2016	6.87%
Samarwickrama et al. [11]	Sri Lanka	2017	7.14%
Present study	North Indian	2021	15%

The variations of roots of the formation of the median nerve have been documented worldwide. However, morphometric analysis has been reported seldomly. It is postulated that the extra-thin roots may not be significant. In contrast, iatrogenic injury to the unknown thick extra roots may carry clinical implications according to the root value of that root. Such injury may cause atypical symptoms that may not correspond with the usual medial nerve injury symptoms. We observed that the thickness of the additional roots varied up to 5 mm. We also observed two unique variations that have not been reported previously. The first variation had an extra “Y” shaped root with innervation from both medial and lateral cords forming the supernumerary root to the median nerve. The second variation had the lateral cord supernumerary root arising from the lateral aspect of the lateral cord that merged with the median nerve after its formation. Further large sample studies will probably explore the actual incidence of these unique variations.

The current study’s limiting factors are the fewer specimens examined and the lack of gender differentiation information. The cadaveric specimens were available and studied as parts rather than the full cadavers. In addition, the study is a cadaveric one and can have differences with the real-life morphometric variations of the median nerve.

## Conclusions

We observed supernumerary roots of varying morphology contributing to the median nerve formation. These variations should be considered during the administration of regional anesthesia and the management of brachial plexus injuries. Further large sample sizes and multi-region studies will help better understand these variations and their actual incidence.
